# Simultaneous learning of directional and non-directional stimulus relations in baboons (*Papio papio*)

**DOI:** 10.3758/s13420-022-00522-8

**Published:** 2022-04-21

**Authors:** Thomas F. Chartier, Joël Fagot

**Affiliations:** 1 Laboratoire de Psychologie Cognitive, CNRS and Aix-Marseille Université, UMR 7290, Bâtiment 9 Case D, 3 place Victor Hugo, 13331 Marseille, Cedex 3 France; 2 Station de Primatologie, CNRS-Celphedia, UPS 846, 13790 Rousset-sur-Arc, France

**Keywords:** Associative symmetry, Directionality, Stimulus equivalence, Baboons, Primate cognition

## Abstract

While humans exposed to a sequential stimulus pairing A-B are commonly assumed to form a bidirectional mental relation between A and B, evidence that non-human animals can do so is limited. Careful examination of the animal literature suggests possible improvements in the test procedures used to probe such effects, notably measuring transfer effects on the learning of B-A pairings, rather than direct recall of A upon cuing with B. We developed such an experimental design and tested 20 Guinea baboons (*Papio papio*). Two pairings of visual shapes were trained (A1-B1, A2-B2) and testing was conducted in a reversed order, either with conserved pairings (B1-A1, B2-A2) or broken ones (B1-A2, B2-A1). We found baboons’ immediate test performance to be above chance level for conserved pairings and below chance level for broken ones. Moreover, baboons needed less trials to learn conserved pairings compared to broken ones. These effects were apparent for both pairings on average, and separately for the best learned pairing. Baboons’ responding on B-A trials was thus influenced by their previous A-B training. Performance level at the onset of testing, however, suggests that baboons did not respond in full accordance with the hypothesis of bidirectionality. To account for these data, we suggest that two competing types of relations were concomitantly encoded: a *directional* relation between A and B, which retains the sequential order experienced, and a *non-directional* relation, which retains only the co-occurrence of events, not their temporal order.

**Supplementary Information:**

The online version contains supplementary material available at 10.3758/s13420-022-00522-8.

## Introduction

Humans presented with word pairings in a serial order A-B seem to spontaneously form the reverse association B → A alongside the trained one A → B. This has been researched in the Paired Associate studies of the 1950s–60 s, and is evidenced by transfer effects, when learning of new word pairings that include either word A or word B is influenced by the untrained, backward B → A association (e.g., Harcum, 1953; Murdock, 1956), but also, upon presentation of word B, by direct recall of word A (e.g., Feldman & Underwood, 1957; Stoddard, 1929). Notwithstanding a debate on the equal strength of both directions of the association (Asch & Ebenholtz, 1962; Houston, 1964; for a review, see Ekstrand, 1966), such results were overall robust and authors interpreted them as human subjects readily forming bidirectional mental relations between word A and word B after a serial exposure.

From an evolutionary perspective, it matters to know whether non-human animals can also form bidirectional relations between stimuli presented serially, or if this capacity is unique to humans, and maybe related to language. Relevant information on this issue can be found in the associative learning literature, based on both Pavlovian and operant conditioning protocols.

### Pavlovian conditioning protocols

One first way to investigate the bidirectionality of associations after a forward pairing A-B is the use of complex^1^ Pavlovian conditioning protocols. Following *backward second-order conditioning* in rats (i.e., exposure to A-US (unconditioned stimulus) then to A-B), Cole et al. (1995, Exp. 1) have reported conditioned responding to A but also to B, the stimulus not paired with the US (A and B being 5-s auditory stimuli and the US aversive^2^). Postulating that a backward untrained association B → A existed, i.e., that training with forward pairings A-B created a bidirectional association A ↔ B, could account for such results in that the chained association B → A → US would give B its new properties, but Cole et al. (1995) interpreted their data differently. Based on the influence of varying the A-US interval, they argued instead for integration of stimulus timing across both training phases, resulting in a *temporal map* (cf. Matzel et al., 1988) representing all three stimuli, and in B becoming predictive of the US. Cole et al. (1995) swapped training phases (i.e., A-B then A-US) in their second experiment. This procedure, called *backward sensory preconditioning*, led to the same results and interpretation.

Backward sensory preconditioning was also employed by Ward-Robinson and Hall (1996), who, though with a different theory in mind, similarly argued against an explanation in terms of a backward association B → A. Using a long auditory stimulus as A and a short visual stimulus as B, they too found conditioned responding to B in rats, but did not favor a temporal map account, as B would not predict the US given their temporal parameters. Their complementary Experiment 3 allowed these authors to propose that a representation of B, originating from the A-B pairing, was later activated by A during the A-US pairing, allowing this evoked representation to directly become associated with the US and acquire response-eliciting properties.

A similar interpretation to that in Ward-Robinson and Hall (1996) had been given by Holland (1981) in a rat experiment involving two forward pairings: first A-US^+^ between an auditory stimulus A and an appetitive US, then A-US^−^ between A and an aversive US. After this, responses to the US^+^ were partly inhibited. The author likewise excluded the chained association account (US^+^ → A → US^−^), which postulates a bidirectional association A ↔ US^+^, proposing instead that A activated a representation of US^+^ during the second pairing, which led to joint activation of US^+^ and US^−^ representations, hence to devaluation of the US^+^. Other rat conditioning experiments reported by Holland (1990) support this notion that a CS (conditioned stimulus) such as A can activate the representation of a US following forward CS-US pairings. Yet, in this “representation-mediated” interpretation of conditioning, only US representations can be activated,^3^ and only in a forward manner, which seems insufficient to explain the more standard, *forward* sensory preconditioning (A-B then B-US), in which B would not activate any representation of A during B-US pairing, hence no A → US association would ensue; this shortfall could bring us back to assuming a temporal encoding of the stimuli.

As these three examples illustrate, there is no unified account of the directionality of associations formed in Pavlovian conditioning experiments using sequential pairings. As a result, some current Pavlovian models assume that associations are bidirectional (e.g., *HeiDI*; Honey et al., 2020) while some do not (e.g., *A-learning*; Ghirlanda et al., 2020).

### Operant conditioning protocols

Perhaps an easier way to compare associative mechanisms between humans and non-humans is the operant procedure of Conditional Matching-to-Sample (CMTS; Cumming & Berryman, 1965), which avoids response-eliciting stimuli such as USs, and can be implemented in a reasonably comparable way across species (e.g., in humans and monkeys (Sidman et al., 1982) or in humans and pigeons (Navarro & Wasserman, 2020)). CMTS protocols always involve a training phase followed by a test phase. In training trials, a *sample* stimulus A is presented first, followed by *comparison* stimuli, namely a stimulus B arbitrarily associated with A presented alongside one or several non-associated stimuli. While correct selection of B and incorrect selection of other stimuli are differentially reinforced, two or more associations are learned, A1 → B1, A2 → B2, etc., generally between visual stimuli or sometimes auditory ones. Typical test trials assess bidirectionality by direct recall – with B as sample, correct selection of A among comparison stimuli constitutes evidence that the B → A association emerged during training – and are conducted as *probes*: they are non-reinforced, to exclude any learning of B → A, and interspersed within reinforced forward trials, to maintain responding.

Bidirectionality studies involving CMTS procedures are generally related to *stimulus equivalence*, a notion presented by Hull (1939) as the acquired capacity of several stimuli to evoke the same behavior in an animal, but given its current meaning by Sidman and his colleagues. According to Sidman and Tailby (1982), stimulus relations learned in a CMTS task are conditional ones such as “If A is presented as sample, then I should answer B” (“If A, then B”), but these can furthermore be considered equivalence relations if a participant spontaneously displays three types of untrained, or *derived*, relations: reflexivity (“If A, then A”), transitivity (“If A, then C,” having learned “If A, then B” and “If B, then C”), symmetry (“If B, then A,” having learned “If A, then B”). In CMTS studies on stimulus equivalence, the question of bidirectionality is thus reduced to this third property of *derived symmetry*, or simply, symmetry.

It is commonly accepted that humans readily show symmetry in CMTS studies, hence non-verbal associations would also be bidirectional – but we have recently warned against a general lack of rigor in these demonstrations (Chartier & Fagot, 2022), which should caution the reader to put this consensus in perspective. In non-humans, symmetry certainly has limited empirical support, as is apparent from two thorough reviews by Lionello‐DeNolf: out of 40 studies, the author considered that 16 produced no evidence whatsoever, and she retained only seven with unambiguously positive results and no obvious alternative explanation (Lionello-DeNolf, 2009, Table 1; Lionello‐DeNolf, 2021, Table 1). Among these seven studies, two involved sea lions (Kastak, Schusterman, & Kastak, 2001; Schusterman & Kastak, 1993), five involved pigeons (Campos et al., 2014; Frank & Wasserman, 2005; Swisher & Urcuioli, 2013, 2015; Urcuioli, 2008), and noticeably none involved primates.

Importantly, we note that all seven studies involved direct recall (probe trials), and did not limit their training procedure to A-B, but included additional pairings referred to as *identity* (A-A and B-B: all of them except Campos et al., 2014), *dual oddity* (A1-A2, A2-A1, B1-B2, B2-B1: Campos et al., 2014), or *partial symmetry* (B-A for a subset of stimuli: Schusterman & Kastak, 1993), and aimed at familiarizing animals with a variable temporal position of stimuli. Thus, at least pigeons and sea lions can probably form bidirectional associations following forward pairings in such complex CMTS protocols, but it remains unknown whether they can do so spontaneously, i.e., after only A-B trials. In contrast, at least one CMTS study with mere A-B training in humans has reported derived symmetry in the very first test trials (Arntzen & Haugland, 2012). Hence, though humans appear more prone to encode bidirectional associations in CMTS procedures than non-humans, it would be crucial to know whether symmetry can emerge in non-humans after A-B training alone.

### Transfer effects in conditional matching-to-sample (CMTS) studies on symmetry

It turns out that three studies using only A-B training, and the alternative testing approach of transfer effects, did find weak evidence for symmetry in pigeons (Hogan & Zentall, 1977) or capuchin monkeys (D’Amato et al., 1985; Soares Filho et al., 2016). The strategy used to reveal bidirectionality was to train participants with two or more A-B pairings, and subsequently have them learn reversed pairings that were either consistent (B1-A1, B2-A2, etc.), or inconsistent (e.g., B1-A2, B2-A1, etc.). Faster learning for the consistent pairings was taken as evidence that the B → A association was already present after training and transferred to the second phase, hence that forward training A-B creates bidirectional associations A ↔ B. We believe that various shortcomings may have prevented clearer demonstration of symmetry in these studies, and that transfer effects deserve further exploration.

Hogan and Zentall (1977) separated 12 pigeons in two test groups, consistent and inconsistent, and found a difference in immediate test performance apparent in the first 28, but not 48, test trials. They found, however, no faster overall learning in the first group and concluded there was a “*minimal*” (p. 13) strength of the derived B → A association. They did not run a control for stimulus effects and their design did not allow comparison between both conditions in each participant. D’Amato et al. (1985) tested six capuchin monkeys successively with consistent and inconsistent pairings, and reported immediate test performance indicative of symmetry in two monkeys, but their test was too short (24 trials) to allow reversed pairings to be learned until criterion, and no binomial tests were applied to compare performance to chance level. Doing so (on six test trials per pairing) reveals that responses of one subject only, Dagwood, achieved statistical significance, and only in one session. Moreover, stimulus preference may have accounted for the results. Last, Soares-Filho et al. (2016) trained one capuchin monkey on two pairings tested with consistent reversals, then on two new pairings tested with inconsistent reversals. A difference in learning length for reversed pairings suggested symmetry; however, no control was provided for the possibility that inconsistent pairings were intrinsically harder to learn than consistent ones (e.g., due to stimulus choice), nor for an effect of condition order (e.g., the second test may take longer simply due to boredom).

Finally, three further investigations of symmetry through transfer effects can be mentioned: Richards (1988), who found a slightly faster learning for consistent pairings in 20 pigeons, but only in a condition where X-A training with new stimuli X was added; Velasco et al. (2010), who reported a weak symmetry effect in one out of four pigeons when including partial symmetry training; and Lionello-DeNolf and Urcuioli (2002), who found no symmetry in 12 pigeons, even when adding identity training.

Together, these six reports encouraged us to make a new attempt at demonstrating symmetry in non-humans with transfer effects. The aim of the present study, conducted in 20 Guinea baboons (*Papio papio*) and using a CMTS procedure, was to examine transfer effects on consistent versus inconsistent reversed pairings, while correcting for shortcomings of previous studies, notably: no additional training; both consistent and inconsistent conditions tested in all participants; control for the order of conditions; control of stimulus effect thanks to randomization across a large number of participants. We quantify here transfer effects on the reversed pairings in two ways: first test block performance (immediate transfer) and length of learning to criterion (global transfer). We provide responses on individual initial test trials, as a complementary measure.

## Methods

### Subjects

Twenty Guinea baboons (*Papio papio*) participated in the experiment, including 14 females and 6 males ranging from 4 to 24.5 years old. The participants come from two social groups of 19 and 6 individuals maintained in two outdoor enclosures, with a total of 700 m^2^, at the CNRS Primatology Station, Rousset-sur-Arc, France. Animals were provided with water ad libitum and fed daily at 4 pm. All participants had previously been exposed to touch screen-involving experiments, including Conditional Matching-to-Sample tasks.

### Apparatus

From the enclosure, the baboons had free, permanent access to computerized testing rooms containing a total of 14 Automated Learning Devices for Monkeys (ALDM, described in detail in Fagot & Bonté, 2010, and Fagot & Paleressompoulle, 2009). Each ALDM is equipped with a 19-inch touch screen, a food dispenser, and an electronic reader that identifies each baboon through the Radio Frequency Identifier (RFID) implanted in its arm. Whenever a baboon enters an ALDM of its choice, the system continues the experiment at the point where it last was, ensuring experimental continuity irrespective of ALDMs. A customized program written with E-Prime software (Professional V. 2.0, Psychology Software Tools, Pittsburgh, PA, USA) controlled the experiment, implementing automatized operant conditioning.

### Stimuli

**Fig. 1:**
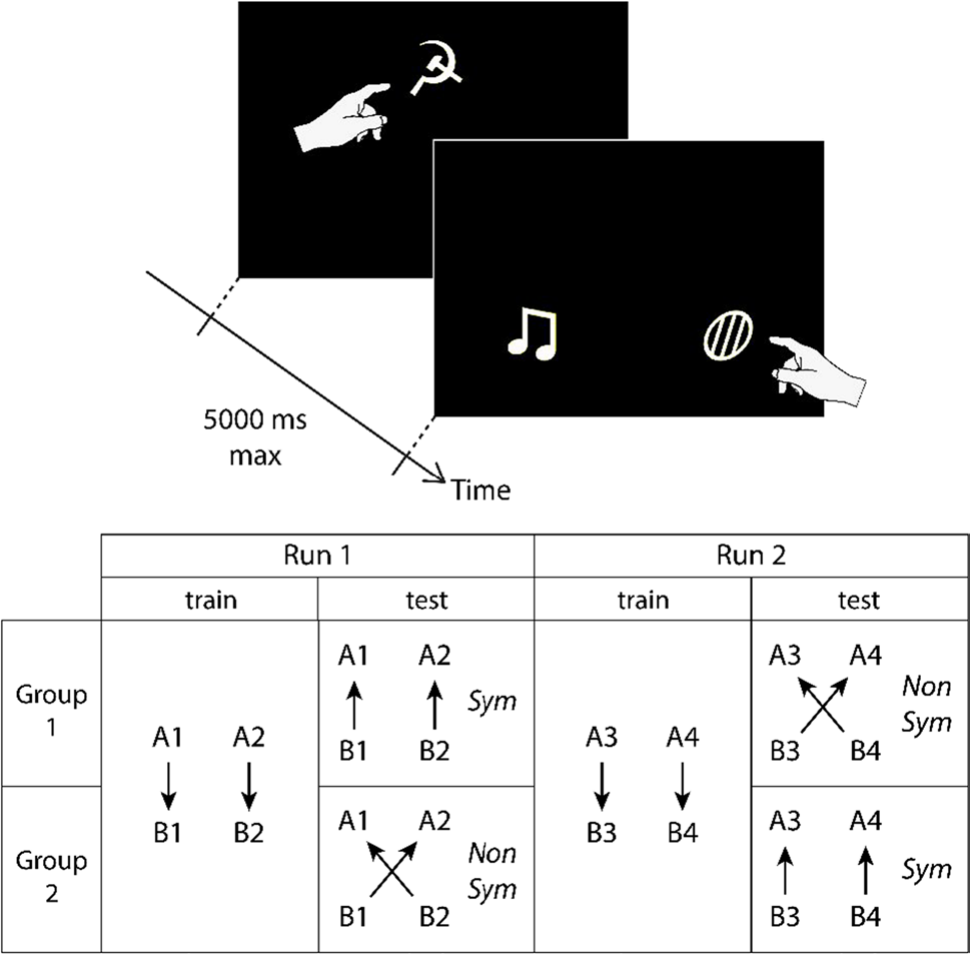
General procedure. Top: For each trial, the subject had to touch the sample stimulus and then one of the two comparison stimuli presented on the screen (the schematic hand depicts the trained behavior). Bottom: Subjects were assigned to two groups, and each group was exposed to two successive runs of training and testing using two new pairings of stimuli for each run. The Sym and NonSym conditions were presented in a counterbalanced order for the two groups and two runs

Eight visual shapes were used for the experiment. Individual stimuli, white and approximately 130 × 130 pixels in size, were presented on a 1,024 × 768 pixel black background. Examples can be seen on Fig. 1.

### General procedure

Baboons were tested in a zero-delay CMTS task. A trial was initiated when a baboon approached its RFID-implanted hand from the touch screen. Each trial began with a sample stimulus being displayed in a top-center position on the screen. The baboon had to touch this stimulus, after which the stimulus was instantaneously removed and followed (zero-delay procedure) by two comparison stimuli displayed in the bottom-left and bottom-right locations (balanced throughout the experiment, see Fig. 1). Touching the correct comparison stimulus cleared the display and delivered a few grains of dry wheat as reward, which ended the trial; touching the incorrect one was followed by a 3-s green screen acting as negative reinforcer, which ended the trial. A total of 5,000 ms was allowed for responding to each display, an absence of response resulting in trial abortion and re-presentation. The inter-trial interval was set to a minimum of 3 s and could be longer depending on the baboon’s willingness to continue. By trial-and-error, the baboons thus progressively learned pairings between sample and comparison stimuli. The experiment consisted of two consecutive runs of training-then-test. The same trial procedure was used for all four phases.

### Training phase

Each of the two runs of training was conducted with four stimuli, hence every participant was exposed to all individual stimuli from our set of eight. A training run consisted of learning two stimulus pairings in a forward direction (e.g., A1-B1, A2-B2) and was organized in blocks of 40 trials. Within each block, pairings appeared 20 times each, presented in a randomized order. Training blocks were presented until the subject reached a performance of at least 80% correct trials for each pairing within one block, i.e., 16 correct answers for each pairing. When this criterion was fulfilled, the test started. Comparison stimuli were always B1 and B2 in the first run, B3 and B4 in the second run. Importantly, the stimulus taking the role of A1, A2, B1, B2, A3, A4, B3, or B4 was randomized across participants, to ensure that variations in performance would not be related to a stimulus’ actual visual identity. Our naming of stimuli in Fig. 1 (e.g., B3) and in the remainder of the article thus refers to the stimulus’ procedural role. As a result, a given couple of visual shapes was never presented as a pairing to more than three individuals in a given run.

### Test phase

Each training was immediately followed by a test phase in which the same stimuli were presented, but stimuli trained as samples were now comparisons and vice versa. Comparison stimuli were thus always A1 and A2 in the first run, A3 and A4 in the second run (see Fig. 1). Two test conditions were introduced: either the reversal conserved the trained pairings (new trials B1-A1, B2-A2: this condition is referred to as the *Sym condition*) or it broke them (new trials B1-A2, B2-A1: *NonSym condition*). To control for order effects, subjects had been assigned to one of two groups: Group 1, composed of 12 individuals, received the Sym test in the first run and the NonSym test in the second run; Group 2, composed of eight individuals, received the NonSym test in the first run and the Sym test in the second run (Fig. 1). In this way, a within-subject comparison of both conditions was possible. Imbalanced group size was due to four non-reported subjects who did not complete the experiment within the time allowed, otherwise groups were balanced in sex and age as much as possible. Apart from stimulus order and pairing, all test trials were identical to training ones and were therefore also differentially reinforced. The test was likewise organized in blocks of 40 randomized trials, 20 for each pairing, and proceeded until a final performance of 80% for each pairing within one block was reached. In short, the test can be considered a re-learning experiment, with previously trained pairings either reversed (Sym) or cross-reversed (Non-Sym).

### Statistical analyses

Two main dependent variables were analyzed: number of blocks to reach criterion in a training or test phase, and average performance on the first block of a training or test phase. When analyzing training phases, repeated-measures ANOVAs were used to determine the effect of Group, Age group, Sex as between-subject factors, and Run as within-subject factor. We analyzed age as a discrete variable by assigning subjects to four age groups: 0–72 months (five subjects), 73–144 months (eight subjects), 145–216 months (five subjects), more than 217 months (two subjects). When analyzing test phases, repeated-measures ANOVAs were used to determine the effect of Group, Age group, Sex as between-subject factors, and Condition as within-subject factor. Group and Age could not be included in the same ANOVA due to an imbalance between groups (only Group 1 was represented in Age group 4: > 217 months), hence instead of conducting a four-way ANOVA we first conducted a Sex by Group by Condition ANOVA and then an Age group by Condition ANOVA.

To conveniently express the changes in learning length between training and test, for each subject and separately for each run a ratio was calculated between the number of test blocks and the number of training blocks, and taken to the logarithm base 2. This log-ratio has several advantages. By computing relative variations between training and test, the ratio normalizes learning length across individuals, which could otherwise obscure intra-individual differences between training and test. The log regularizes data and symmetrizes distributions: a log-ratio of + 1 means the test was two times longer than the training, -1 means it was two times shorter.

For statistical significance, the conventional threshold of 0.05 was used for all tests and indicated as follows in the figures: *n.s.* = *p* > 0.05, * = *p* < 0.05, ** = *p* < 0.005, *** = *p* < 0.0005, **** = *p* < *0.00005.* Effect sizes are reported as either η_p_^2^ for ANOVAs, Cohen’s *d* for t-tests, and the Z statistic divided by the square root of the sample size n for the Wilcoxon test. Two-tailed t-tests were used. Post hoc comparisons in ANOVAs were performed using Tukey’s *Honestly Significant Difference* test. All analyses were performed using the R software version 4.0.3.

## Results

### Learning of A-B pairings (training)

The baboons took between 2 and 19 days to complete both runs of the experiment. The mean number of blocks to criterion was 64.1 (SEM = 16.8) in the first training and 49.9 (SEM = 8.6) in the second training (individual learning curves for all participants are shown in the Online Supplementary Material (OSM)). Using the number of training blocks as the dependent variable, we first inspected whether training performance was different between the first and second run of the experiment, and whether it was influenced by group, sex or age group. A repeated-measures ANOVA for Group (1 vs. 2) by Run (1 vs*.* 2) revealed no main effect of Group, Run, and no significant interaction between Group and Run (all *ps* > 0.30). The Sex by Run ANOVA revealed no main effects of Sex, Run, and no significant interaction between Sex and Run (all *ps* > 0.46). The Age by Run ANOVA revealed no main effects of Age group, Run, and no significant interaction (all *ps* > 0.19). Hence, the variables of group, age, sex, and run had no detectable influence on general learning performance.

Since immediate test performance, defined as performance averaged over the first test block, was to be analyzed subsequently, effects of transfer needed to be separated from effects of group or of task practice. We therefore verified that mean performance over the first training block was comparable for each group and run. A Group by Run ANOVA confirmed that no main effects of Group, Run, or significant interaction were present (all *ps* > 0.17). In addition, none of the four mean performances differed from chance level in a t-test (all *ps* > 0.29). Initial performance in both training phases was thus homogeneous and at chance level.

### Were the A-B pairings equally well learned?

**Fig. 2:**
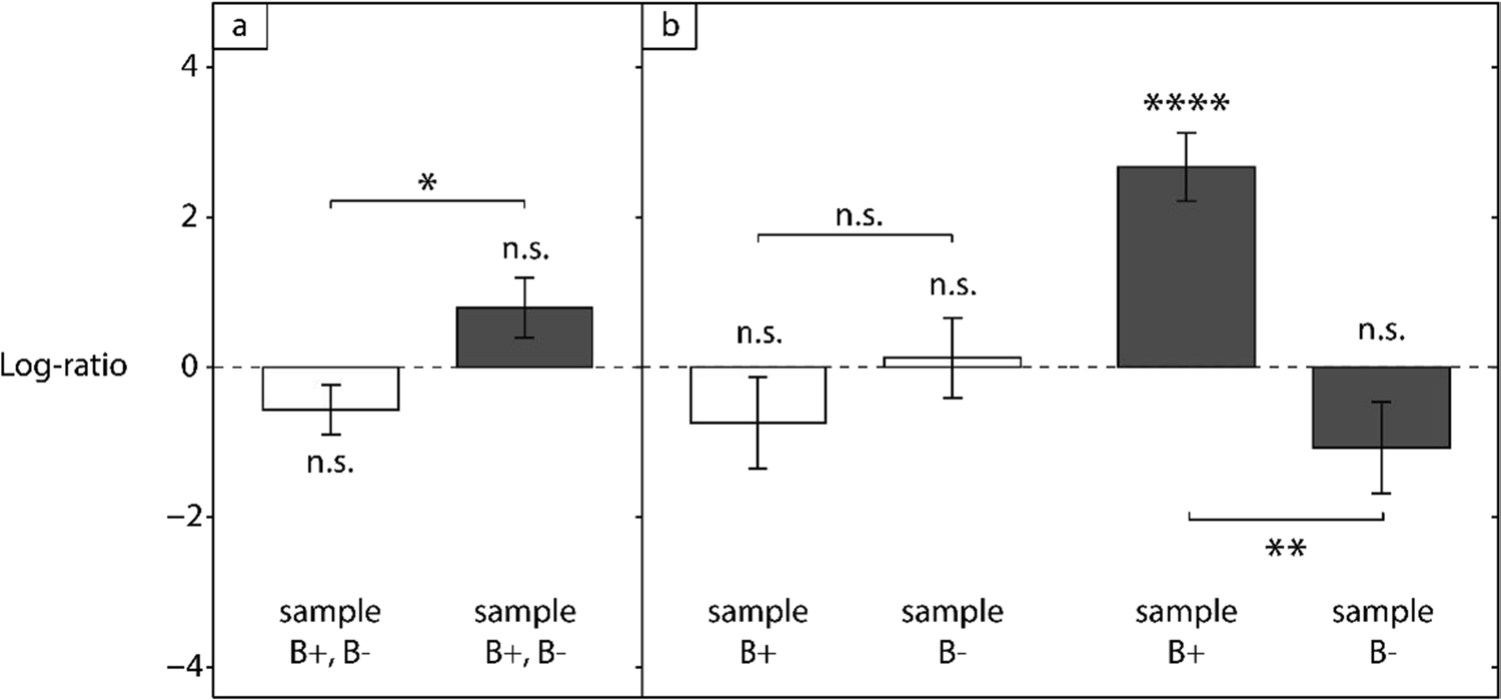
Comparisons of changes in number of blocks between training and test, expressed as log-ratios, together for both pairings (**a**) or separately for B + /B- as sample in test (**b**). Log-ratios are averaged across the 20 subjects and plotted as bars with standard errors. Empty bars refer to the Sym condition, dark grey bars to the NonSym condition. The dashed line indicates chance-level performance

**Fig. 3:**
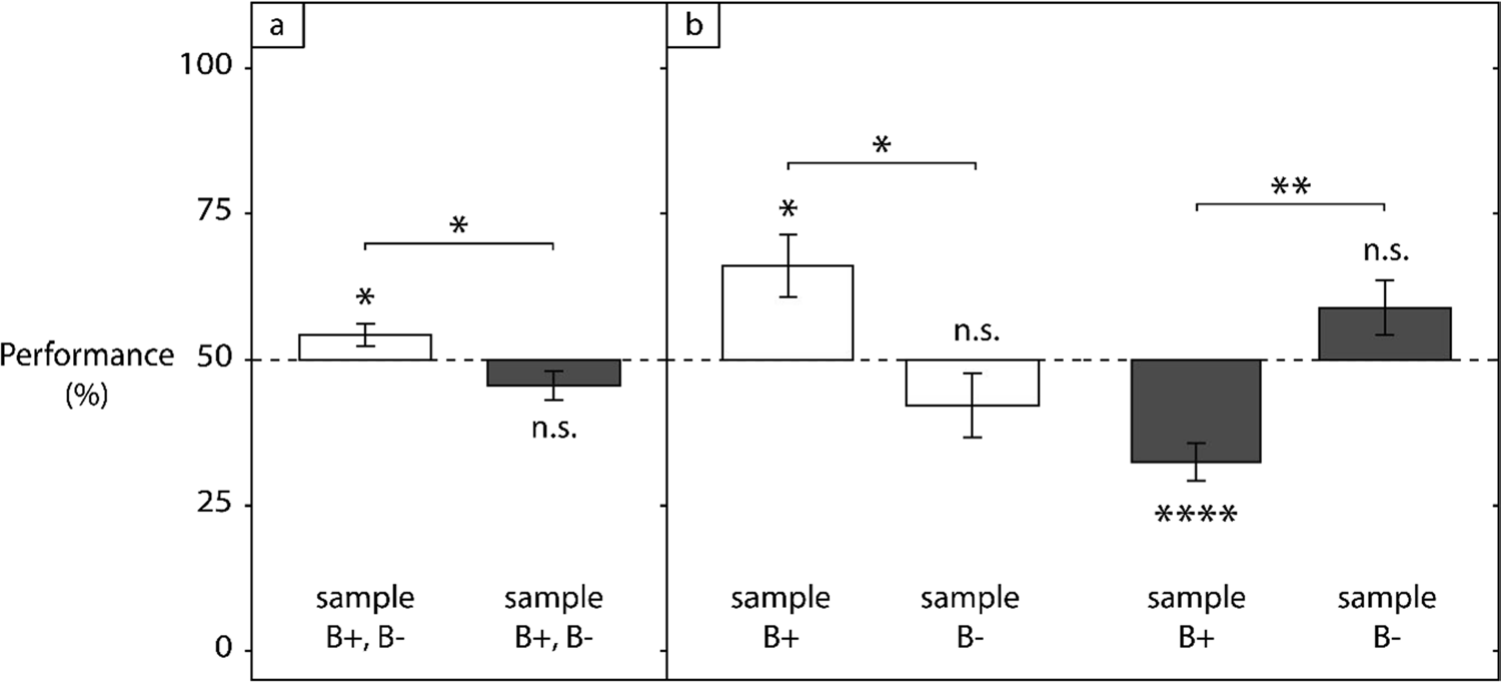
Comparison of immediate test performance between conditions, together for both pairings (**a**) or separately for B + /B- as sample in test (**b**). Performance is averaged across the 20 subjects and plotted as bars with standard errors. Empty bars refer to the Sym condition, dark grey bars to the NonSym condition. The dashed line indicates chance-level performance

Because baboons tend to acquire pairings of items in a successive, cumulative manner (see Dépy et al., 1997), we reasoned that one of the two trained pairings may have been better learned than the other. If this had been the case, stronger changes in number of blocks, resulting in larger amplitude of log-ratios, would be observed for the best pairing. We thus examined the last ten blocks of training (i.e., 200 trials per pairing) for each subject and each training, and found that one pairing had systematically had more blocks at the 80% performance criterion than the other, namely a difference of three blocks on average. This value was significantly greater than 0 as assessed by a Wilcoxon test, Z(40) = 408, *p* < 0.00005, with a large effect size, r = 0.61. This difference between pairings allowed us to identify, for each subject and each training, a “pairing + ” (designated as A^+^-B^+^) having more blocks at criterion during the last ten blocks, therefore assumed to be better learned, and a “pairing-” (designated as A^−^-B^−^), the other one, assumed to be less well learned. For example, if one individual had learned A2-B2 better than A1-B1 in the first training, A1 was now designated as A^−^, A2 as A^+^, B1 as B^−^, B2 as B^+^; and similarly for the second training (in one single case of equal number of training blocks at criterion, A^+^-B^+^ was defined as having the highest mean performance over the last ten blocks). Note that the actual stimuli forming A^+^-B^+^ and A^−^-B^−^ necessarily varied across individuals, due to the random permutations of stimulus identity applied between individuals (see 5). Test performance data reported further below include separate analyses for A^+^-B^+^ and A^−^-B^−^, indicated by “B^+^ as sample” or “B^−^ as sample” in Figs. 2 and 3.

### Learning of B-A pairings (test)

#### Number of blocks to criterion (global transfer)

The mean number of blocks to criterion was 42.0 (SEM = 9.6) for the test in Sym condition and 77.1 (SEM = 13.1) for the test in NonSym condition (individual learning curves for all participants and phases are shown in the OSM). Number of blocks was analyzed here using as dependent variable a log-ratio calculated between training and test (see 5). If subjects had formed symmetrical relations during training, one would expect an effect of Condition, that is, an easier learning during test in Sym compared to NonSym condition, resulting in less test blocks in Sym as well as a lower log-ratio. A Sex by Group by Condition ANOVA indeed revealed only a main effect of Condition, *F*(1,16) = 5.72, *p* < 0.05, η_p_^2^ = 0.17. No main effect of Sex or Group was found, and no reliable interaction of the higher level either (all *ps* > 0.26). An Age group by Condition ANOVA confirmed that the effect of Condition was reliable, *F*(1,16) = 3.46, *p* < 0.05, η_p_^2^ = 0.11, but revealed no reliable effect of Age group or interaction (both *ps* > 0.29). Since both groups differ mainly in the order of conditions (Sym then NonSym, vs. NonSym then Sym), the absence of Group effect suggests that the order of conditions had no influence on the results, hence data from both groups were pooled and subsequent log-ratio analyses included all 20 individuals.

Regarding the effect of condition, baboons learned the reversed pairings faster in the Sym condition compared to the NonSym condition, as hypothesized (paired t-test, *t*(19) = -2.49, *p* < 0.05, *d* = 0.56, see Fig. 2a): the mean log-ratio was -0.57 in the Sym condition, indicating a 33% decrease in number of blocks between training and test, and + 0.80 in the NonSym condition, indicating a 73% increase. The difference from zero of both values was close to significance (*t*(19) = -1.71, *p* = 0.10, *d* = 0.38 and *t*(19) = -1.71, *p* = 0.06, *d* = 0.45, respectively, Fig. 2a). This shows that compared to training, learning during test lasted for less blocks with reversed pairings, but for more blocks with cross-reversed pairings.

Next, we analyzed the potential effect of A^+^-B^+^ and A^−^-B^−^ on test length. Because pairings are broken in the NonSym condition in test, log-ratio data in Fig. 2b are presented according to which stimulus, B^+^ or B^−^, was presented as sample. We therefore calculated, for each condition and each sample, the number of blocks needed to first reach 80% performance for the corresponding pairing. Before taking the log, this number was divided by its corresponding number of training blocks, i.e., that of A^+^-B^+^ for pairings in the Sym condition and that of A^−^-B^−^ for pairings in the NonSym condition. A Condition (Sym vs. NonSym) by Sample (B^+^ vs. B^−^) ANOVA revealed a main effect of Condition, *F*(1,19) = 6.74, *p* < 0.05, η_p_^2^ = 0.049, and of Sample, *F*(1,19) = 8.19, *p* < 0.05, η_p_^2^ = 0.081, as well as a significant interaction, *F*(1,19) = 9.07, *p* < 0.01, η_p_^2^ = 0.18. In the Sym condition, log-ratios did not differ between trials using B^+^ and B^−^ as samples, as assessed by Tukey’s test, *t*(38) = -1.07, *p* > 0.1, *d* = 0.47, but in the NonSym condition they did strongly differ, *t*(38) = 4.91, *p* < 0.005, *d* = 1.42 (Fig. 2c). T-tests revealed that only the log-ratio for trials using B^+^ as a sample in the NonSym condition significantly differed from zero, *t*(19) = 5.87, *p* < 0.00005, *d* = 1.31. Hence, while no significant effect was observed when testing with trials using B^−^ as sample, a marked effect was observed with B^+^ trials, though only in the NonSym condition where the number of blocks increased by a factor of about 6.3 (mean log-ratio ≈ 2.67).

#### Performance on the first test block (immediate transfer)

Results on immediate test performance are reported in Fig. 3. Regarding this factor, we expected to find an above-chance level in the Sym Condition, indicative of an initial facilitation for responses to conserved pairings, and a below-chance level in the NonSym Condition, indicative of the opposite effect for responses to broken pairings. A Sex by Group by Condition ANOVA revealed only a main effect of Condition, *F*(1,16) = 5.72, *p* < 0.05, η_p_^2^ = 0.17, but not of Sex, Group and no significant interactions (all *ps* > 0.11). As for number of blocks, the absence of group effect suggests that the order of conditions did not influence the results, hence data from both groups were pooled and subsequent immediate performance analyses concerned all 20 individuals. An Age group by Condition ANOVA confirmed that the effect of Condition was reliable, *F*(1,16) = 14.0, *p* < 0.005, η_p_^2^ = 0.30, but revealed no reliable effect of Age group or significant interaction (both *ps* > 0.06). Regarding the effect of condition, baboons had a better immediate test performance in the Sym compared to the NonSym condition, with mean performances of 54.1% and 45.6%, respectively, the first value being significantly different from chance, *t*(19) = 2.25, *p* < 0.04, *d* = 0.50, the second one close to significance, *t*(19) = 1.80, *p* < 0.10, *d* = 0.40, and both values significantly different from each other, *t*(19) = 2.60, *p* < 0.02, *d* = 0.58 (Fig. 3a). Hence, immediate test performances did show an influence of the previous training, in the direction expected, which suggests a facilitation for reversed pairings, and a hindrance for cross-reversed ones.

Analyzing pairings separately, we expected a more pronounced effect with B^+^ as sample. A Condition (Sym vs. NonSym) by Sample (B^+^ vs. B^−^) ANOVA revealed a main effect of Condition, *F*(1,19) = 6.77, *p* < 0.05, η_p_^2^ = 0.040, but not of Sample, *p* > 0.80, and a significant interaction, *F*(1,19) = 13.10, *p* < 0.005, η_p_^2^ = 0.26. Performance differed reliably between B^+^ and B^−^ as sample in both conditions, as assessed by Tukey’s test, *t*(38) = 3.08, *p* < 0.05, *d* = 0.98 in the Sym condition, and *t*(38) = -4.59, *p* < 0.005, *d* = 1.45 in the NonSym condition (Fig. 3b). For B^+^ as sample, performance in the Sym condition was at 66% and significantly above chance level, *t*(19) = 2.98, *p* < 0.01, *d* = 0.67, while in the NonSym condition performance was at 32.5% and significantly below chance, *t*(19) = -5.38, *p* < 0.001, *d* = 1.20. For B^−^ as sample, however, neither performance differed significantly from chance. These results showed that for B^+^ as sample, immediate test performance was substantially altered by the previously trained pairings, which led to improved responding in Sym, and to systematic errors in NonSym.

### Further evidence of symmetry for A^+^-B^+^

**Fig. 4:**
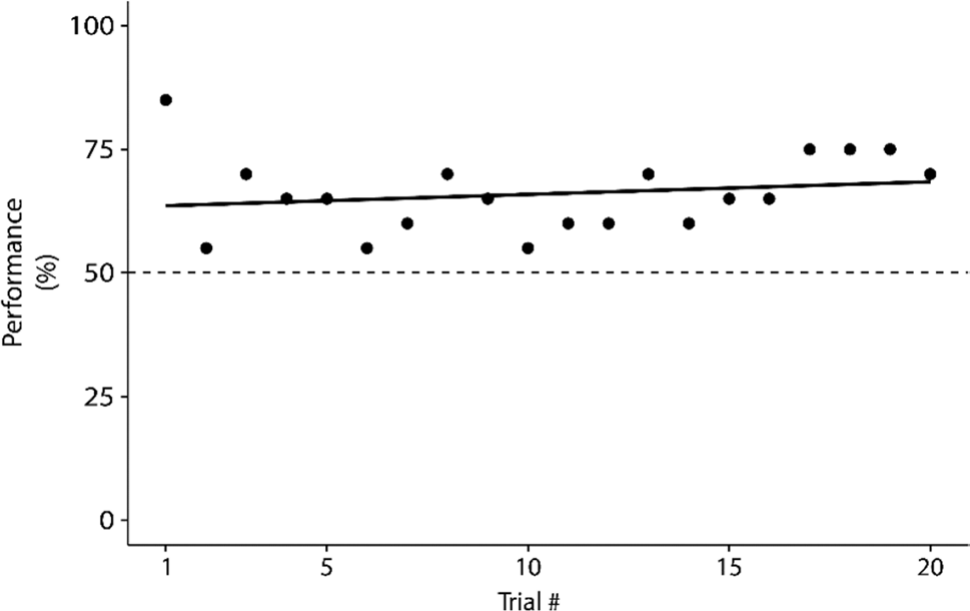
Performance averaged across the 20 subjects, for individual trials of the first test block, with B + as sample. The dashed line indicates chance-level performance; the solid line is the regression line, R^2^ =  0.037, F(1,18) =  0.688, *p* = 0.42, y = 63.3 + 0.256x

We further scrutinized potential symmetry effects, concentrating on A^+^-B^+^. Since all test trials are differentially reinforced, above-chance performance in Sym condition (see Fig. 3b) could be due to rapid learning, rather than to a true transfer of the knowledge acquired during training. In this case, performance could start at chance level and rapidly increase in the remainder of the first test block, resulting in an overall block performance significantly above chance. To exclude this possibility, we examined individual trial performance in the Sym condition on the first test block, that is, 20 trials for B^+^ as sample (Fig. 4). The strongest indication of symmetry comes from the first test trial, where we found that 17 out of 20 subjects responded correctly, a value significantly above chance (*p* < 0.005) in the exact binomial test. This trial has a particular scientific meaning, because it is the only one where participants have not yet received feedback related to the new pairing. First trial data thus assess the most uninfluenced, spontaneous answers, which here revealed symmetry. Moreover, the regression slope for these 20 points did not significantly differ from zero (β_1_ = 0.256, *p* = 0.42), which speaks against the idea of a rapid learning of the new pairings from scratch. Arguably, one may want to remove the first trial from this regression, due to its special status. Doing so makes the regression slope slightly different from zero (β1 = 0.630, *p* = 0.019), which could indicate that some learning takes place, though at a slow pace. In sum, both first- and 20-trial data suggest that the previously learned A^+^-B^+^ pairing had influenced initial responding in test, leading to more correct answers than expected by chance alone.

**Fig. 5:**
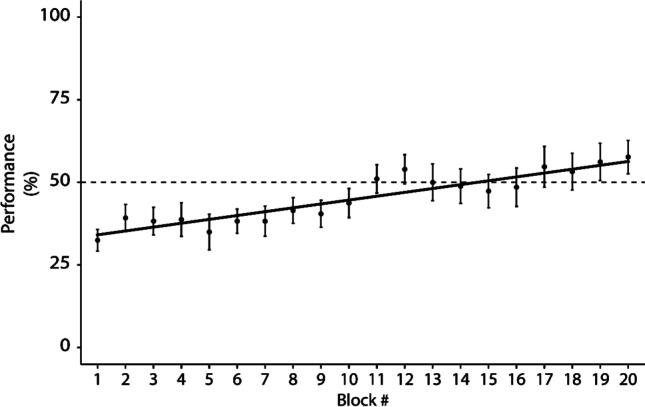
Block performance averaged over the 20 subjects, for the first 20 test blocks in the NonSym condition, with B + as sample. Error bars represent standard errors. The number of subjects is 20 up to block #10, 19 for blocks #11 and 12, 18 for block #13, and 17 from block #14. The dashed line indicates chance-level performance; the solid line is the regression line, R^2^ = 0.85, F(1,18) = 104.9, *p* = 6.2e-9, y = 32.8 + 1.198x

In support of this conclusion, it should be noted that the below-chance immediate test performance with B^+^ as sample in the NonSym condition (see Fig. 3b) could by no means be accounted for by rapid learning. Interestingly, we found that starting from 32.5% in test block 1, about 14 blocks were needed on average to reach 50% performance in the NonSym condition, i.e., 280 trials with B^+^ as sample, according to the regression shown in Fig. 5. This persisting hindrance cannot be due to a stimulus effect, as stimulus identity varied across individuals. Instead, it suggests that responses consistent with the now-incorrect pairing B^+^-A^+^ were difficult for subjects to inhibit. This constitutes a strong argument in favor of a symmetrical relation for A^+^-B^+^.

## Discussion

### Evidence for symmetry

In this study involving a CMTS protocol where baboons successively learned A-B pairings (training) and B-A pairings (test) with visual stimuli, we looked at two complementary indicators of symmetry: immediate test performance and test length. The latter likely depends on the former, as an improved initial performance in test should make it easier to reach the criterion. For both indicators, our data revealed symmetry-consistent effects mainly due to A^+^-B^+^, the pairing that was best learned during training.

In the absence of symmetry, the B-A pairings presented in test would have been treated as new ones and the length of learning, though likely shorter than in A-B training due to stimulus familiarity, would have been equal between Sym and NonSym. Here, this was not the case. With both pairings considered together, test length was shortened in Sym, by about one-third, while it was lengthened in NonSym, by about three-quarters. This effect was even stronger when A^+^-B^+^ was considered alone, though only in the Nonsym condition, with a more than sixfold increase (Fig. 2). B-A learning was thus facilitated in Sym and impeded in NonSym. This clearly suggests baboons had somehow also formed the reverse relation B → A during training.

In the absence of symmetry, one would still expect immediate test performance to be at a 50% chance level. When both pairings were considered together, it was at 54.1% in Sym and 45.6% in NonSym. For A^+^-B^+^ alone, these performances were even at 66% and 32.5% respectively (Fig. 3) and reliably different from chance-level. This second indicator confirms that the trained relations are likely to have influenced initial B-A learning, because baboons started with either an advantage or a disadvantage, depending on the condition.

We excluded the possibility that improved immediate test performance in Sym could be explained by a rapid learning. The advantage for B^+^ as sample was indeed present from the very first trial (Fig. 4), the response to which is the only one unaffected by reinforcement. Inspection of the below-chance performance observed for B^+^ as sample tested in the NonSym condition further contradicts the existence of rapid learning, as this deterioration persists for nearly 300 trials (Fig. 5). We can also exclude the possibility that these altered immediate test performances were due to a stimulus preference for A^+^ in both the Sym and the NonSym conditions: had this been the case, systematic errors would have made performance for B^−^ as sample significantly below chance in Sym and above chance in NonSym, while here no significant difference was found (Fig. 3). Furthermore, the fact that stimulus shapes and order of conditions were counterbalanced across subjects rules out a possible effect of these factors. In our study, the symmetry effects took the form of a learning facilitation for consistent pairings and a learning hindrance for inconsistent ones. Importantly, these results were obtained in the absence of partial symmetry training, identity training, or dual oddity training. As such, they constitute an important demonstration of symmetry after unidirectional training, and show the validity of transfer approaches in this field of research.

The fact that despite our having 20 subjects these effects were only moderate when analyzing both stimulus pairings together illustrates why symmetry could have been missed in previous monkey studies with few individuals (e.g., D’Amato et al., 1985; Sidman et al., 1982). Our finding of stronger symmetry effects for the best learned stimulus pairing shows the relevance of separate pairing analysis in these studies, and of ensuring the best possible learning. We believe that symmetry effects will be more convincingly brought to light when combining measurements of immediate and global transfer, as we did here. Our study also suggests that considering data only from one or a few individuals can overshadow the existence of bidirectional associations.

### Why was training performance only partially transferred?

Though our baboons had formed more than a strictly unidirectional relation during training, we note that their behavior in test was not in full accordance with symmetrical relations. Had this been the case, B^+^-A^+^ performance in the first test block should have been comparable to A^+^-B^+^ performance in the last training block (around 80%), while here it was only at 66%. Similarly, baboons should have reached the performance criterion within the first test block, which none of them did. Could such partial transfer be a sign of partially symmetrical relations, as D’Amato and co-authors proposed, when they suggested that relations formed after unidirectional exposure “*became increasingly bidirectional over evolution, approaching symmetry in humans*” (D’Amato et al., 1985, p. 46)? This could explain why close evolutionary relatives to humans, such as capuchin monkeys in their experiment or baboons in ours, do not fully show symmetry. However, in our opinion a notion of partial bidirectionality is not satisfactory, since if upon cuing with B, A is not always retrieved, retrieval is nevertheless possible, hence the backward direction B → A is encoded. One should rather think of this in terms of imperfect, probabilistic retrieval. An association is bidirectional or is not, but when it is, one direction may be partially accessible.

One interpretation for partial accessibility is that in non-human animals, the functional stimulus, i.e., the stimulus actually processed, includes both spatial position and temporal position of the stimulus during training, not simply its visual identity (for an overview of this issue, see Frank & Wasserman, 2005, pp.148–149). The prominence of spatial information in stimulus pairing studies has been documented in pigeons (Lionello-DeNolf & Urcuioli, 2002; Swisher & Urcuioli, 2015), rats (Iversen, 1997), and non-human primates (Iversen et al., 1986), including Guinea baboons (Fagot et al., 2018). In our experiment, both A and B stimuli had new locations during test, a clear potential source of performance decrement. We had chosen not to remedy this by presenting stimuli at randomized spatial positions, since preliminary unpublished testing in our laboratory had shown that such variability, even restricted to three locations, considerably lengthens training and reduces performance in baboons. The second issue, temporal position, has been mainly investigated in pigeons (e.g., Frank & Wasserman, 2005; Urcuioli, 2008). When non-human participants are not familiarized *before test* with B also coming first and A also coming second, this may affect their matching performance when reversed B-A trials are introduced. To cancel this surprise effect, authors have proposed to also train A-A and B-B pairings (identity training, (Frank & Wasserman, 2005; Sidman et al., 1982), or X-A and Y-B pairings (Richards, 1988, Exp. 2; Hayes, 1989, p. 387), or even some of the B-A pairings (partial symmetry training, e.g., Schusterman & Kastak, 1993). Here, we avoided these approaches because they bring additional stimulus relations potentially interfering with the object of interest. Therefore, surprise due to a new temporal position of the stimuli remains a plausible explanation for baboons’ performance decline at the onset of testing.

In sum, if functional stimuli were indeed three-faceted ones in our experiment, two of these facets (i.e., spatial and temporal position, but not visual identity) were changed during testing, hence baboons were effectively exposed to *partially* new stimuli and had to learn *partially* new relations, which resulted in the *partial* transfer of performance observed here. Let us note that if temporal position is part of the stimulus, then pairings cannot be reversed without altering the functional stimuli. Hence, it seems normal that symmetry revealed by direct recall in non-humans has been best documented so far in CMTS studies including additional training such as identity training, not after mere A-B training.

### On two coexisting types of stimulus associations

The issue at hand in the present article is commonly formulated as follows: “is a stimulus association formed during a forward serial pairing unidirectional or bidirectional?”, with an overall agreement that associations are typically bidirectional in humans but rarely in non-humans. The fact that both possibilities are mutually exclusive is difficult to reconcile with how we process inherently directional and possibly irreversible sequences. For example, having learned to say “good morning,” we do not assume that the backward pairing “morning good” is authorized. Similarly, if strong winds are followed by uprooted trees, one does not expect observation of an uprooted tree to predict strong winds.

To overcome such pitfalls, we would like to propose another line of thinking. Instead of an opposition, we suggest a complementarity between two types of relations that are encoded concomitantly by humans but also by baboons. On the one hand, a *directional* relation between A and B, which respects the sensory evidence accumulated by the animal, retaining an indication of strict unidirectionality if A is always followed by B. On the other hand, a *non-directional* relation, which retains only the information of which stimuli or objects go together in the environment and which ones do not, a relation encoding co-occurrences and not sequences (since A and B would evoke each other here, such relations may perhaps still be qualified as symmetrical, in the sense of mutual retrieval). In our view, unlike envisaged above, visual stimuli in a CMTS procedure would not have three facets but at most two: shape and possibly location. Temporal position would not be encoded as part of the functional stimulus but in a dedicated relation, in addition to a co-occurrence relation. The directional and non-directional relations, possibly interacting, would together form the complete association between A and B.

It is apparent that humans trained with serial pairings A-B are able to encode both types of relation. In CMTS studies, subjects’ spontaneous A answers in B-A test trials (e.g., Arntzen & Haugland, 2012) reveal that they possess information independent of order. But Pavlovian trace conditioning studies show that human subjects do know about the directionality of forward pairings, as correct verbal reports of CS-US temporal relationships demonstrate (e.g., Clark & Squire, 1998). In other words, humans encode co-occurence *and* direction, not co-occurence *rather than* direction. Regarding non-humans, our data show that both encodings may also coexist in CMTS protocols, the directional one being evidenced during training and the non-directional one at the onset of testing, though partially expressed. Whether baboons preferentially access the directional relation, or rather have difficulties in accessing the non-directional one, remains to be decided. In any case, some kind of competition or preference between both could account for the known difficulty to provide experimental proof of symmetry after unidirectional training.

In sum, with our two-process account of stimulus associations, the difference between humans and non-humans observed in all studies mentioned in the introduction would not be a sign of different ways of *encoding* the A-B serial pairing, but a sign of differential *access* to two concomitantly acquired encodings.

### Where does the human/non-human difference come from?

Independently of one’s interpretation of stimulus relations formed in matching protocols, an interspecific difference in how readily backward associations are expressed cannot be denied, and remains to be explained.

One first possible account for this difference could lie at the attentional level. When processing spatial visual information, humans have a stronger attentional focus on global information, in comparison to baboons whose processing is more local (Fagot & Deruelle, 1997). Similarly, humans are more proficient than baboons at retrieving the structural complexity of long visuo-motor sequences, suggesting that they have a more direct access to the globality of the temporal sequences (Rey et al., 2019). In baboons, it can be argued that the weight given to (local) information only accessible at the level of the individual A or B items (such as spatiality) strongly impairs their performance when sequentiality is reversed. This is because such reversals also alter the (local) information that can be retrieved at the level of the individual A or B items. This effect would not be that strong for humans, for which a more global processing of the pair facilitates the detection of non-directional information.

In a different perspective, it can also be proposed that a verbal recoding of stimulus relations, a priori unavailable to non-humans, may be a potent facilitator of bidirectional associations. Humans readily name visual stimuli, even abstract ones not supposed to represent anything (for an example in CMTS studies, see Bentall et al., 1993, Exp. 1). When cued with B after a forward A-B training, and in order to retrieve A, a name previously given to B may well be a more efficient entry than the mere visual trace of stimulus B. Consequently, humans, but not other species, would be proficient in expressing backward associations. Further studies will be required to test the validity of the above two hypotheses.

## Supplementary Information

Below is the link to the electronic supplementary material.

Supplementary file1 (DOCX 346 kb)

## Footnotes

1 Simple Pavlovian protocols typically use *forward* conditioning (CS-US pairings), where the neutral CS intuitively predicts the biologically significant US. *Backward* conditioning (US-CS pairings) assesses whether conditioned responses to the CS develop when this stimulus is paired with, though non-predictive of, the US (see, e.g., Razran 1956), but this protocol only tests one direction of the association, not whether the association is bidirectional. The term “backward” is thus misleading and *backward conditioning* does not inform about *backward associations*. Moreover, as the CS generally elicits no specific response, behavior can only reveal associations towards the US. To avoid such functional asymmetry, at least three stimuli are required, for example 2CS/1US as in second-order conditioning and sensory preconditioning, or 1CS/2US as in Holland (1981).

2 Similar results were reported by Thrailkill and Shahan (2014) in an appetitive protocol.

3 Though Holland (1990, p.125) seems to suggest that the less salient the response-eliciting properties of the US, the more likely the activation by the CS of its representation, which one may extend to conclude that a CS1 could activate a CS2 representation following a forward CS1-CS2 pairing.

## Declarations

### Open practices statement

Raw data, R programs and a few videos of the experiment will be made publicly and freely available online via the Open Science Framework upon publication. The experiment was not pre-registered.

## References

[CR1] Arntzen, E., & Haugland, S. (2012). Titration of limited hold to comparison in conditional discrimination training and stimulus equivalence testing. *The Psychological Record, 62*(2), 243–261. 10.1007/BF03395800

[CR2] Asch, S. E., & Ebenholtz, S. M. (1962). The principle of associative symmetry. *Proceedings of the American Philosophical Society,* 106(2), 135–163. http://www.jstor.org/stable/985378

[CR3] Bentall, R. P., Dickins, D. W., & Fox, S. R. (1993). Naming and equivalence: Response latencies for emergent relations. *The Quarterly Journal of Experimental Psychology,**46*(2), 187–214. 10.1080/14640749308401085

[CR4] Campos, H. C., Urcuioli, P. J., & Swisher, M. (2014). Concurrent identity training is not necessary for associative symmetry in successive matching. *Journal of the Experimental Analysis of Behavior,**101*(1), 10–25. 10.1002/jeab.51 24436073 10.1002/jeab.51 PMC3951194

[CR5] Chartier, T. F., & Fagot, J. (2022). Associative symmetry: a divide between humans and nonhumans? *Trends in Cognitive Sciences*, *26*(4), 286-289. https://doi.org/10.1016/j.tics.2022.01.009 10.1016/j.tics.2022.01.009 35246386

[CR6] Clark, R. E., & Squire, L. R. (1998). Classical conditioning and brain systems: The role of awareness. *Science,**280*(5360), 77–81. 10.1126/science.280.5360.77 9525860 10.1126/science.280.5360.77

[CR7] Cole, R. P., Barnet, R. C., & Miller, R. R. (1995). Temporal encoding in trace conditioning. *Animal Learning & Behavior,**23*(2), 144–153. 10.3758/BF03199929

[CR8] Cumming, W. W., & Berryman, R. (1965). The complex discriminated operant: Studies of matching-to-sample and related problems. In D. I. Mostofsky (Ed.), *Stimulus generalization* (pp. 284–330). Stanford University Press.

[CR9] D’Amato, M. R., Salmon, D. P., Loukas, E., & Tomie, A. (1985). Symmetry and transitivity of conditional relations in monkeys (*Cebus apella*) and pigeons (*Columba livia*). *Journal of the Experimental Analysis of Behavior,**44*(1), 35–47. 10.1901/jeab.1985.44-35 16812425 10.1901/jeab.1985.44-35 PMC1348159

[CR10] Dépy, D., Fagot, J., & Vauclair, J. (1997). Categorization of three-dimensional stimuli by humans and baboons: Search for prototype effects. *Behavioural Processes,**39*, 299–306. 10.1016/s0376-6357(96)00757-7 24897338 10.1016/s0376-6357(96)00757-7

[CR11] Ekstrand, B. R. (1966). Backward associations. *Psychological Bulletin,**65*(1), 50–64. 10.1037/h0022645 5324081 10.1037/h0022645

[CR12] Fagot, J., & Deruelle, C. (1997). Processing of global and local visual information and hemispheric specialization in humans (Homo sapiens) and baboons (Papio papio). *Journal of Experimental Psychology: Human Perception and Performance,**23*(2), 429. 10.1037/0096-1523.23.2.429 9104003 10.1037//0096-1523.23.2.429

[CR13] Fagot, J., & Bonté, E. (2010). Automated testing of cognitive performance in monkeys: Use of a battery of computerized test systems by a troop of semi-free ranging baboons. *Behavior Research Methods,**42*(2), 507–516. 10.3758/BRM.42.2.507 20479182 10.3758/BRM.42.2.507

[CR14] Fagot, J., Malassis, R., & Medam, T. (2018). The processing of positional information in a two-item sequence limits the emergence of symmetry in baboons (*Papio papio*), but not in humans (*Homo sapiens*). *Learning & Behavior,**46*(1), 67–78. 10.3758/s13420-017-0290-1 28779389 10.3758/s13420-017-0290-1

[CR15] Fagot, J., & Paleressompoulle, D. (2009). Automatic testing of cognitive performance in baboons maintained in social groups. *Behavior Research Methods,**41*(2), 396–404. 10.3758/BRM.41.2.396 19363180 10.3758/BRM.41.2.396

[CR16] Feldman, S. M., & Underwood, B. J. (1957). Stimulus recall following paired-associate learning. *Journal of Experimental Psychology,**53*(1), 11. 10.1037/h0048386 13398536 10.1037/h0048386

[CR17] Frank, A. J., & Wasserman, E. A. (2005). Associative symmetry in the pigeon after successive matching-to-sample training. *Journal of the Experimental Analysis of Behavior,**84*(2), 147–165. 10.1901/jeab.2005.115-04 16262184 10.1901/jeab.2005.115-04 PMC1243977

[CR18] Ghirlanda, S., Lind, J., & Enquist, M. (2020). A-learning: A new formulation of associative learning theory. *Psychonomic Bulletin & Review,**27*(6), 1166–1194. 10.3758/s13423-020-01749-0 32632888 10.3758/s13423-020-01749-0

[CR19] Harcum, E. R. (1953). Verbal transfer of overlearned forward and backward associations. *American Journal of Psychology,**66*, 622–625. 10.2307/1418961 13124573

[CR20] Hayes, S. C. (1989). Nonhumans have not yet shown stimulus equivalence. *Journal of the Experimental Analysis of Behavior,**51*(3), 385–392. 10.1901/jeab.1989.51-385 16812585 10.1901/jeab.1989.51-385 PMC1338931

[CR21] Hogan, D. E., & Zentall, T. R. (1977). Backward associations in the pigeon. *American Journal of Psychology,* 90(1), 3–15. http://www.jstor.org/stable/1421635

[CR22] Holland, P. C. (1981). Acquisition of representation-mediated conditioned food aversions. *Learning and Motivation,**12*(1), 1–18. 10.1016/0023-9690(81)90022-9

[CR23] Holland, P. C. (1990). Event representation in Pavlovian conditioning: Image and action. *Cognition,**37*(1–2), 105–131. 10.1016/0010-0277(90)90020-K 2269004 10.1016/0010-0277(90)90020-k

[CR24] Honey, R. C., Dwyer, D. M., & Iliescu, A. F. (2020). HeiDI: A model for Pavlovian learning and performance with reciprocal associations. *Psychological Review,**127*(5), 829. 10.1037/rev0000196 32271046 10.1037/rev0000196

[CR25] Houston, J. P. (1964). S-R stimulus selection and strength of R-S association. *Journal of Experimental Psychology,**68*(6), 563–566. 10.1037/h0048435 14241088 10.1037/h0048435

[CR26] Hull, C. L. (1939). The problem of stimulus equivalence in behavior theory. *Psychological Review,**46*(1), 9. 10.1037/h0054032

[CR27] Iversen, I. (1997). Matching-to-sample performance in rats: A case of mistaken identity? *Journal of the Experimental Analysis of Behavior,**68*(1), 27–45. 10.1901/jeab.1997.68-27 16812849 10.1901/jeab.1997.68-27 PMC1284618

[CR28] Iversen, I. H., Sidman, M., & Carrigan, P. (Trans.). (1986). Stimulus definition in conditional discriminations. *Journal of the Experimental Analysis of Behavior,* 45(3), 297-304. 10.1901/jeab.45-297 10.1901/jeab.1986.45-297 PMC1348240 3711776

[CR29] Kastak, C.R., Schusterman, R.J., & Kastak, D. (2001). Equivalence classification by California sea lions using class-specific reinforcers. Journal of the Experimental Analysis of Behavior, 76, 131–158. 10.1901/jeab.2001.76-131 10.1901/jeab.2001.76-131 PMC1284831 11599636

[CR30] Lionello-DeNolf, K. M. (2009). The search for symmetry: 25 years in review. *Learning & Behavior,* 37(2), 188-203. 10.3758/LB.37.2.188 10.3758/LB.37.2.188 PMC2685068 19380896

[CR31] Lionello-DeNolf, K. M. (2021). An update on the search for symmetry in nonhumans. *Journal of the Experimental Analysis of Behavior,**115*(1), 309–325. 10.1002/jeab.647 33225440 10.1002/jeab.647

[CR32] Lionello-DeNolf, K. M., & Urcuioli, P. J. (2002). Stimulus control topographies and test of symmetry in pigeons. *Journal of the Experimental Analysis of Behavior,**78*(3), 467–495. 10.1901/jeab.2002.78-467 12507015 10.1901/jeab.2002.78-467 PMC1284911

[CR33] Matzel, L. D., Held, F. P., & Miller, R. R. (1988). Information and expression of simultaneous and backward associations: Implications for contiguity theory. *Learning and motivation, 19*(4), 317–344. 10.1016/0023-9690(88)90044-6

[CR34] Murdock, B. B., Jr. (1956). “Backward” learning in paired associates. *Journal of Experimental Psychology,**51*(3), 213. 10.1037/h0044314 13306866 10.1037/h0044314

[CR35] Navarro, V. M., & Wasserman, E. A. (2020). Bidirectional conditioning: Revisiting Asratyan’s ‘alternating’ training technique. *Neurobiology of Learning and Memory,**171*, 107211. 10.1016/j.nlm.2020.107211 32156520 10.1016/j.nlm.2020.107211

[CR36] Razran, G. (1956). Backward conditioning. *Psychological Bulletin,**53*(1), 55. 10.1037/h0044122 13289966 10.1037/h0044122

[CR37] Rey, A., Minier, L., Malassis, R., Bogaerts, L., & Fagot, J. (2019). Regularity extraction across species: Associative learning mechanisms shared by human and non-human primates. *Topics in Cognitive Science,**11*(3), 573–586. 10.1111/tops.12343 29785844 10.1111/tops.12343

[CR38] Richards, R. W. (1988). The question of bidirectional associations in pigeons’ learning of conditional discrimination tasks. *Bulletin of the Psychonomic Society,**26*(6), 577–579. 10.3758/BF03330126

[CR39] Schusterman, R. J., & Kastak, D. (1993). A California sea lion (*Zalophus californianus*) is capable of forming equivalence relations. *Psychological Record,**43*, 823–839. 10.1007/BF03395915

[CR40] Sidman, M., Rauzin, R., Lazar, R., Cunningham, S., Tailby, W., & Carrigan, P. (1982). A search for symmetry in the conditional discriminations of rhesus monkeys, baboons, and children. *Journal of the Experimental Analysis of Behavior,**37*(1), 23–44. 10.1901/jeab.1982.37-23 7057127 10.1901/jeab.1982.37-23 PMC1333116

[CR41] Sidman, M., & Tailby, W. (1982). Conditional discrimination vs. matching to sample: an expansion of the testing paradigm. *Journal of the Experimental Analysis of Behavior,* 37(1), 5–22. 10.1901/jeab.1982.37-5 10.1901/jeab.1982.37-5 PMC1333115 7057129

[CR42] Soares Filho, P. S. D., Silva, A. J., Velasco, S. M., Barros, R. S., & Tomanari, G. Y. (2016). Assessing symmetry by comparing the acquisition of symmetric and nonsymmetric conditional relations in a capuchin monkey. *International Journal of Psychology Research,* 9, 30–39. 10.21500/20112084.2320

[CR43] Stoddard, G. D. (1929). An experiment in verbal learning. *Journal of Educational Psychology,**20*(6), 452–457. 10.1037/h0073293

[CR44] Swisher, M., & Urcuioli, P. J. (2013). Symmetry in the pigeon with sample and comparison stimuli in different locations. *Journal of the Experimental Analysis of Behavior,**100*(1), 49–60. 10.1002/jeab.31 23703090 10.1002/jeab.31 PMC5390681

[CR45] Swisher, M., & Urcuioli, P. J. (2015). Symmetry in the pigeon with sample and comparison stimuli in different locations. II. *Journal of the Experimental Analysis of Behavior*, 104(2), 119–132. 10.1002/jeab.162 10.1002/jeab.162 26216645

[CR46] Thrailkill, E. A., & Shahan, T. A. (2014). Temporal integration and instrumental conditioned reinforcement. *Learning & Behavior,**42*(3), 201–208. 10.3758/s13420-014-0138-x 24879632 10.3758/s13420-014-0138-x PMC4766005

[CR47] Urcuioli, P. J. (2008). Associative symmetry, “anti-symmetry”, and a theory of pigeons’ equivalence-class formation. *Journal of the Experimental Analysis of Behavior,**90*(3), 257–282. 10.1901/jeab.2008.90-257 19070336 10.1901/jeab.2008.90-257 PMC2582203

[CR48] Velasco, S. M., Huziwara, E. M., Machado, A., & Tomanari, G. Y. (2010). Associative symmetry by pigeons after few-exemplar training. *Journal of the Experimental Analysis of Behavior,**94*(3), 283–295. 10.1901/jeab.2010.94-283 21541172 10.1901/jeab.2010.94-283 PMC2972781

[CR49] Ward-Robinson, J., & Hall, G. (1996). Backward sensory preconditioning. *Journal of Experimental Psychology: Animal Behavior Processes, 22*(4), 395–404. 10.1037/0097-7403.22.4.395

