# Correction: Simultaneous learning of directional and non-directional stimulus relations in baboons (*Papio papio*)

**DOI:** 10.3758/s13420-026-00718-2

**Published:** 2026-08-06

**Authors:** Thomas F. Chartier, Joël Fagot

**Affiliations:** 1 Laboratoire de Psychologie Cognitive, CNRS and Aix-Marseille Université, UMR 7290, Bâtiment 9 Case D, 3 Place Victor Hugo, 13331Cedex 3 Marseille, France; 2 Station de Primatologie, CNRS-Celphedia, UPS 846, 13790 Rousset-Sur-Arc, France

**Correction: Learning & Behavior (2022) 51:166–178**

**https://doi.org/10.3758/s13420-022-00522-8**

The source code for the paper was inadvertently omitted:

Raw data, figures, and R analysis scripts are freely available online via the Open Science Framework: https://osf.io/j26f7. The experiment was not pre-registered.

