# Supplementary material for: Simultaneous learning of directional and non-directional stimulus relations in baboons (Papio papio)
Source: Learn Behav. 2022 Apr 21;51(2):166–78. doi: 10.3758/s13420-022-00522-8 (PMC10272242; doi:10.3758/s13420-022-00522-8)

**Simultaneous learning of directional and non-directional stimulus relations in baboons (*Papio papio*).**

Thomas F. Chartier, Joël Fagot

Supplementary material


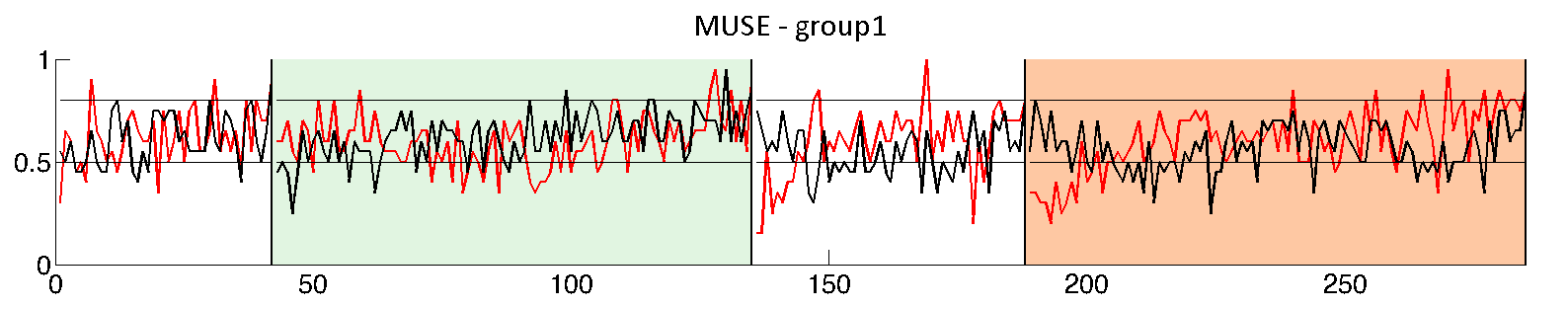

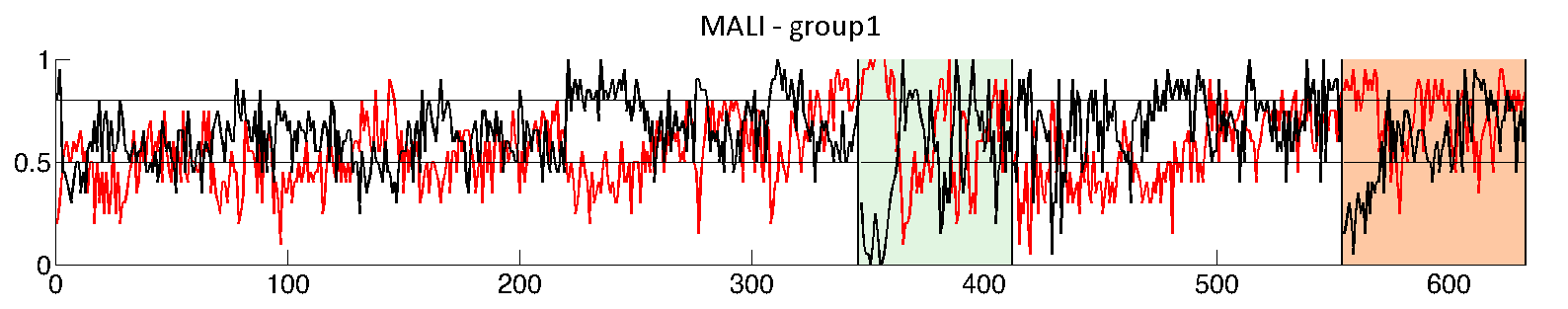

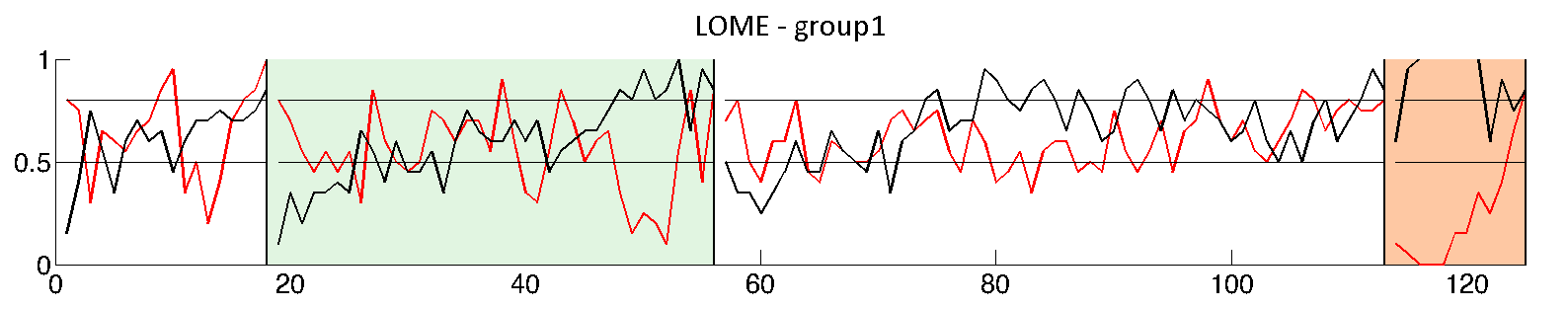
We provide here the individual learning curves for the 4 phases and for all participants. Performance (Y axis, between 0 and 1), *i.e.*, the proportion of correct responses per block of 40 trials, is plotted as a function of number of blocks (X axis). Test phases are indicated by shaded areas, with light green representing Sym condition and light red representing NonSym condition. The corresponding training phases are plotted immediately at their left, on a white background. Performance is plotted separately for both pairs of stimuli, arbitrarily designated as pair #1 (red line) and pair #2 (black line). Note that pairs of stimuli differ between the first two and the last two phases, and are instantiated by different stimuli across participants. The upper horizontal line on each graph indicates the performance criterion set at 0.8 and required to move on to the next phase, the lower one indicates chance-level responding at 0.5.


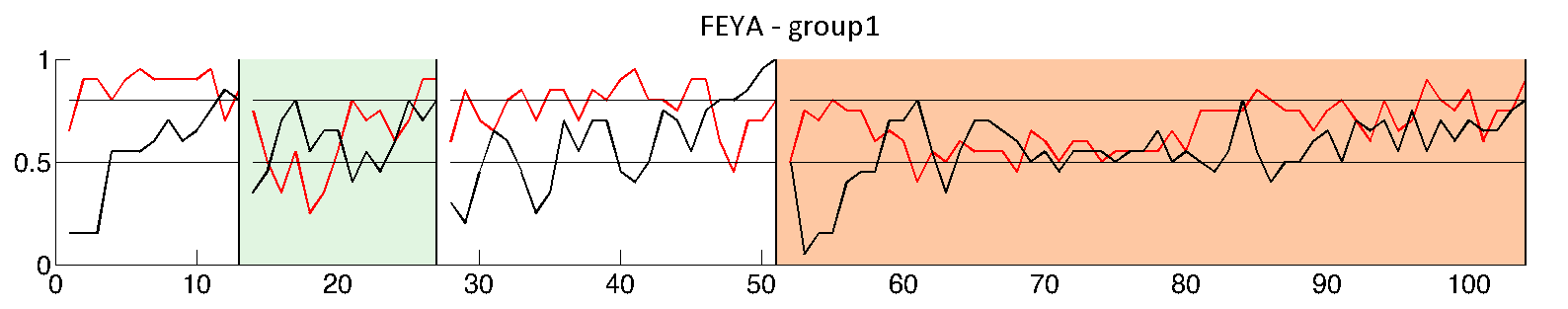

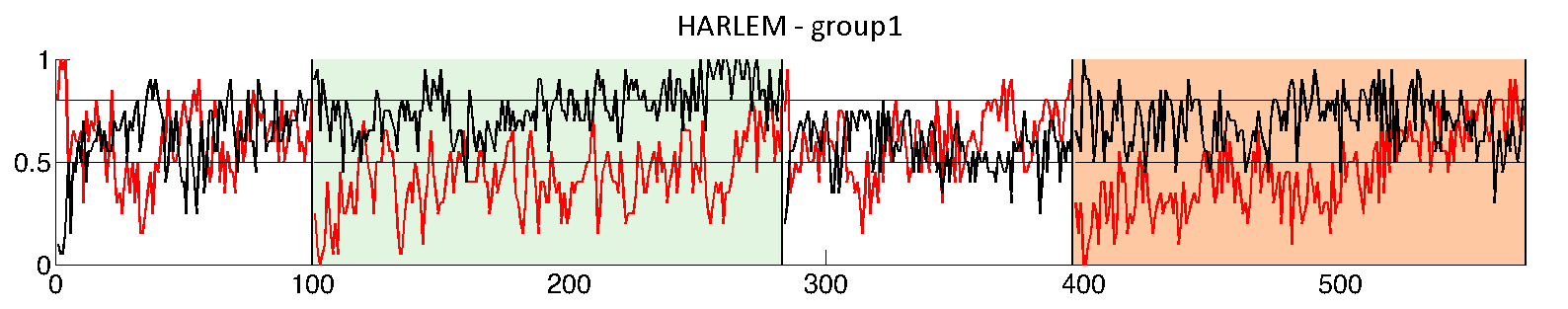

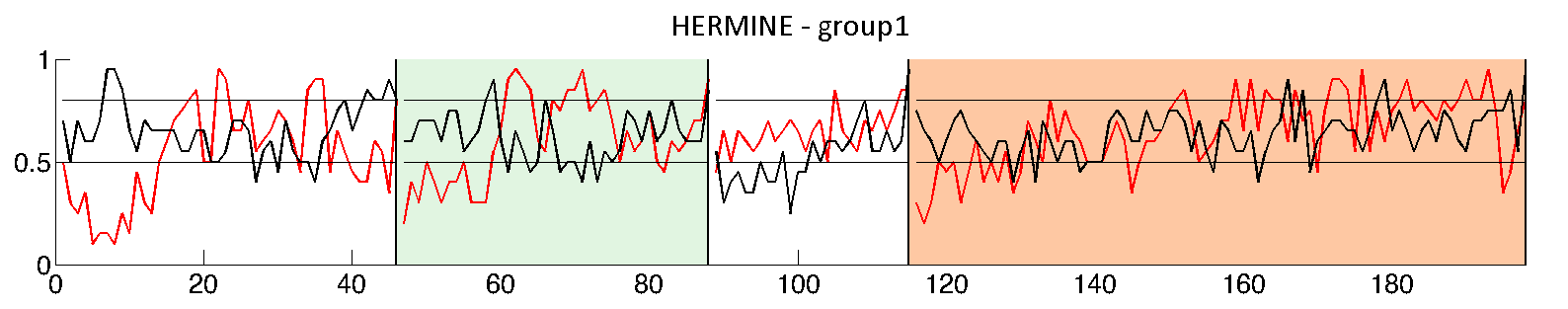

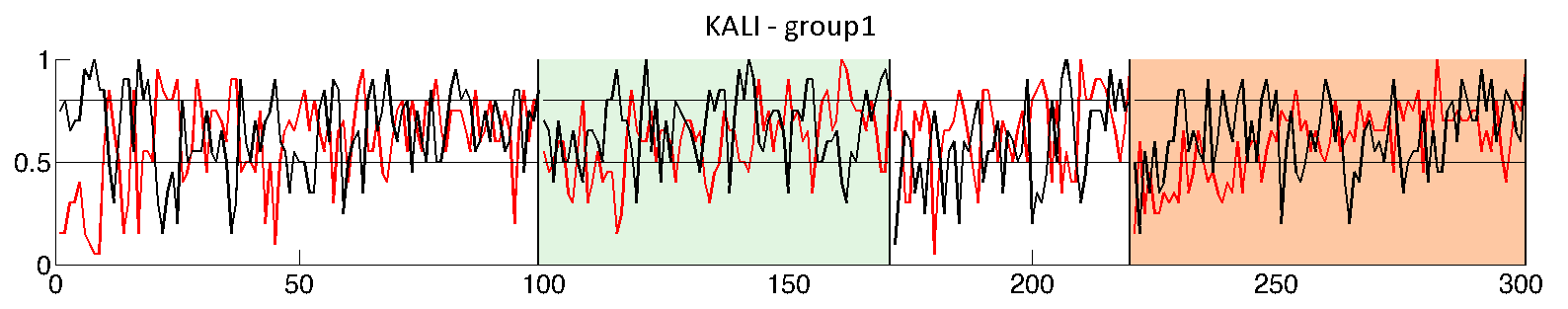


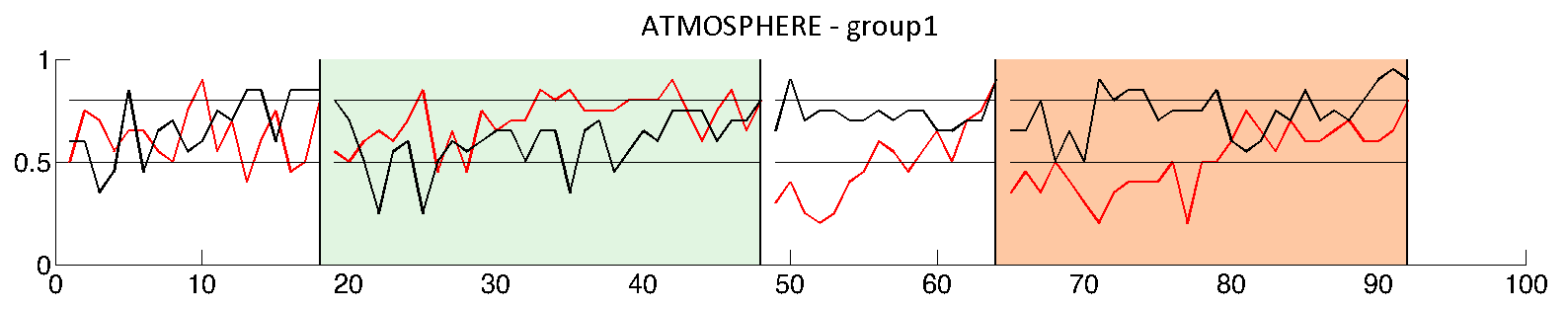

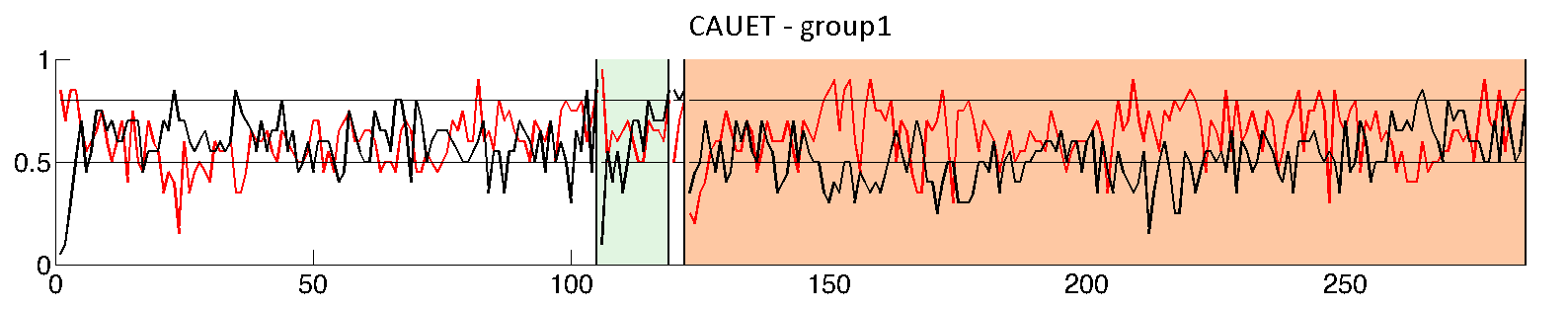

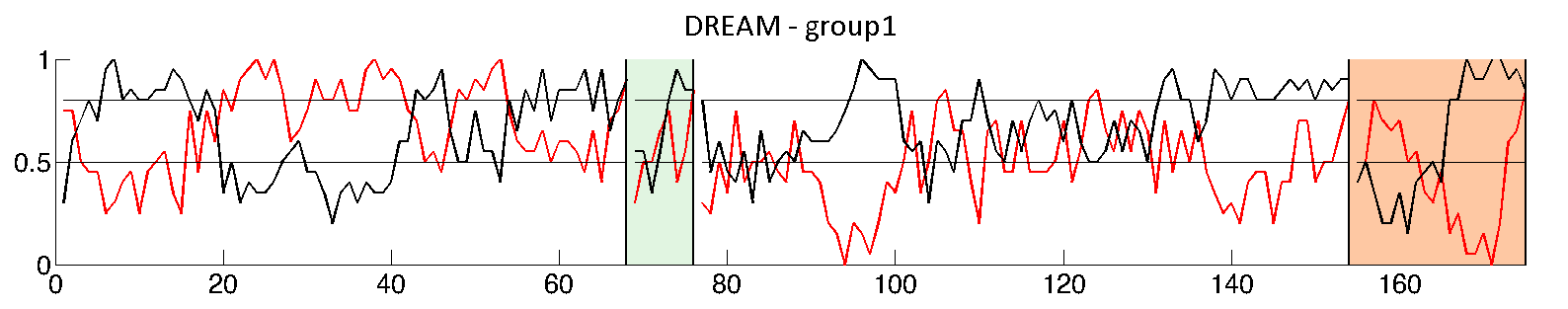

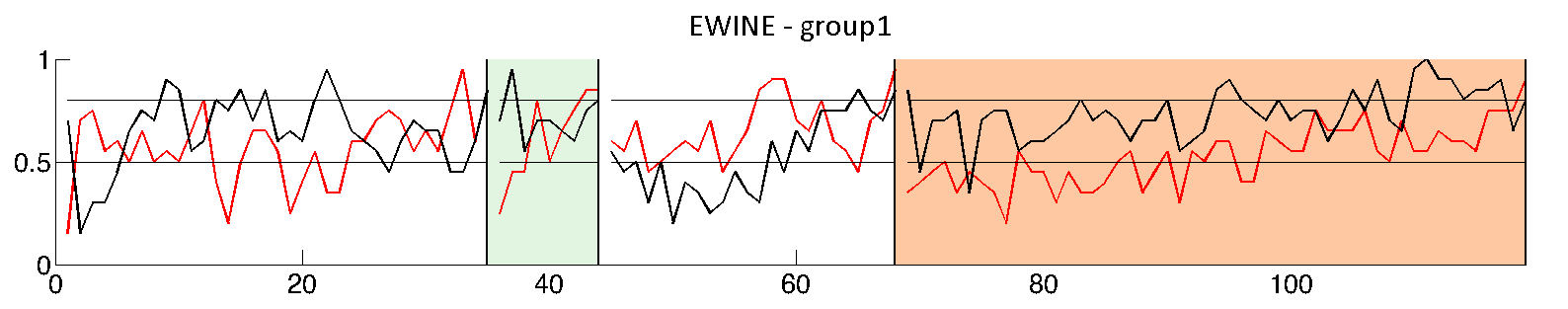


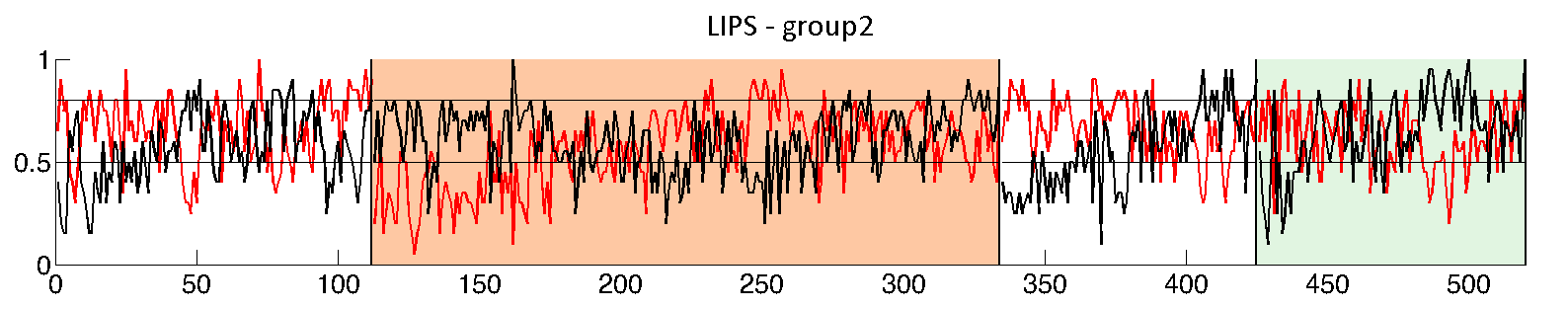

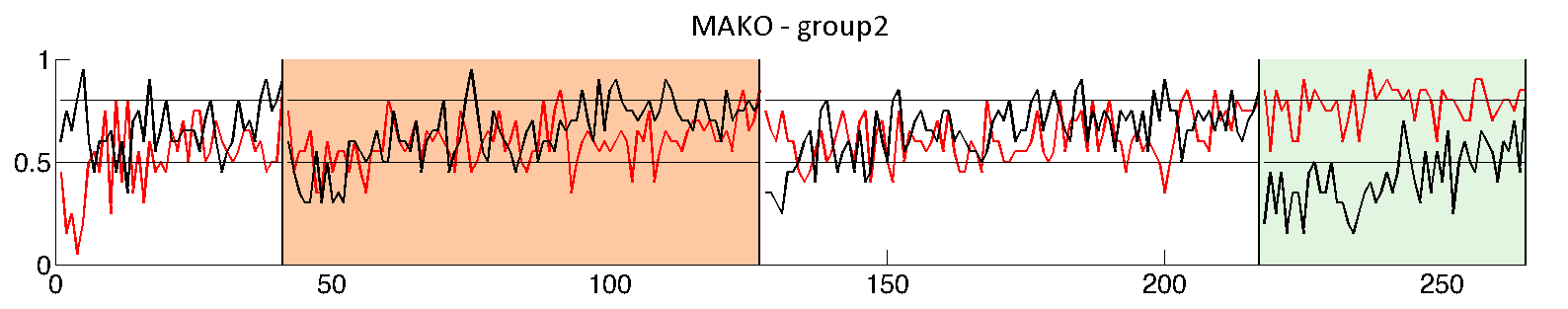

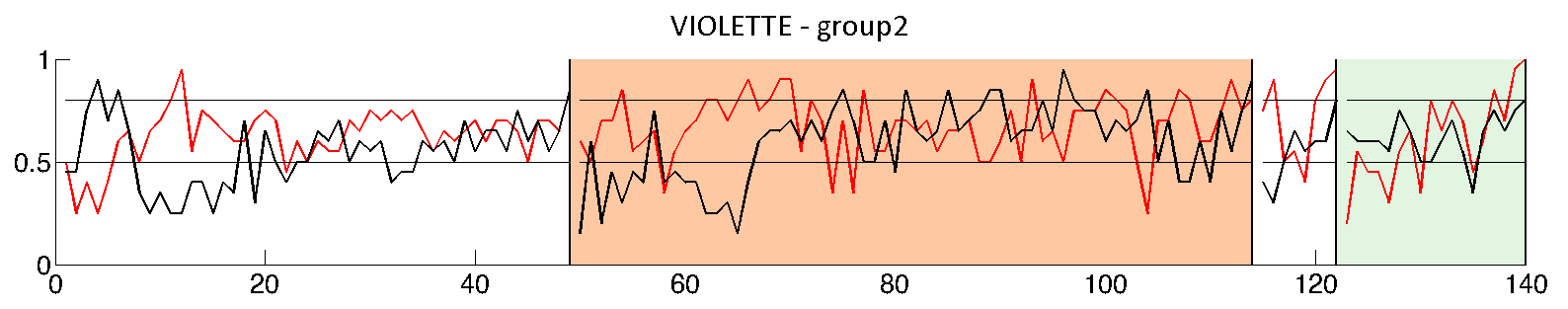

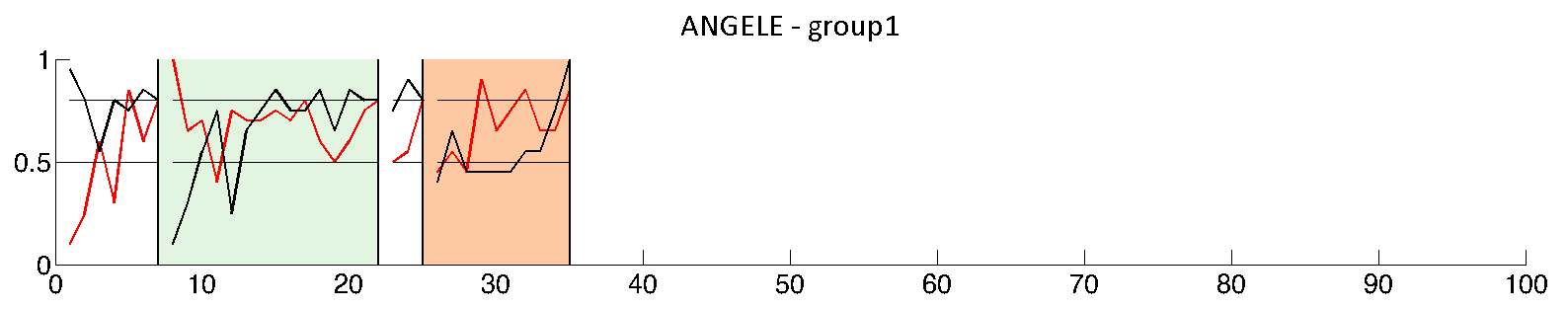


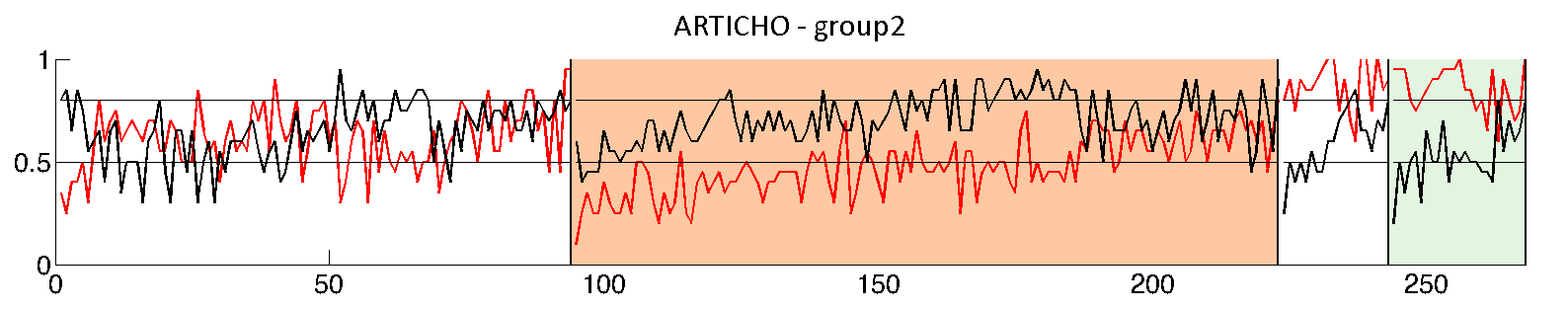

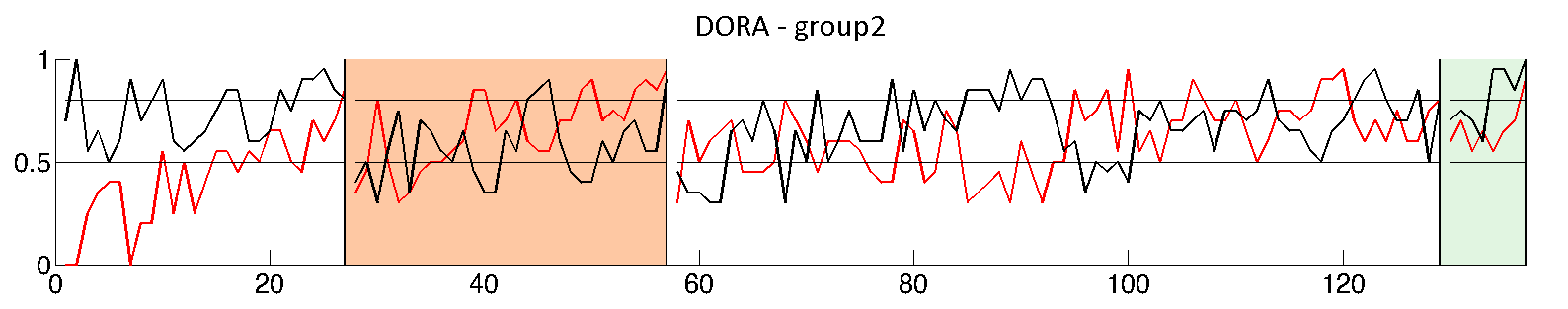

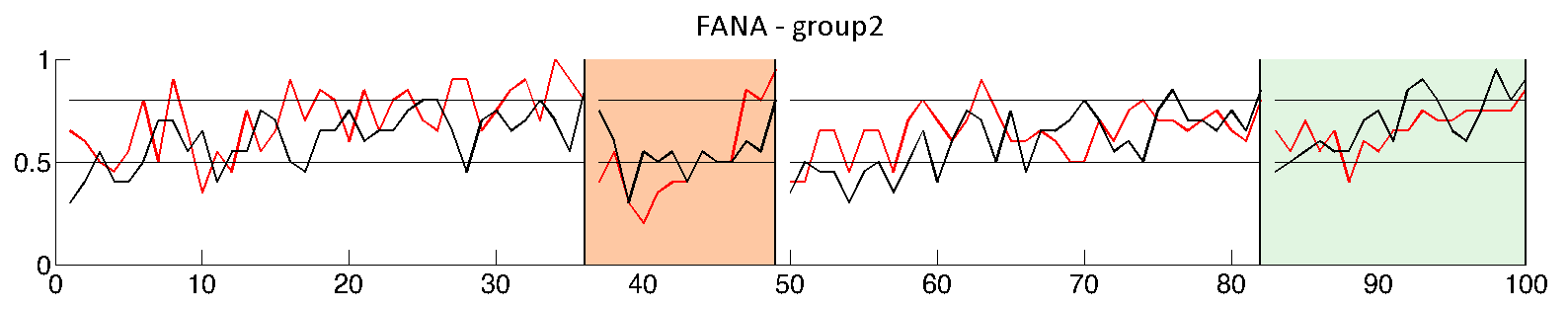

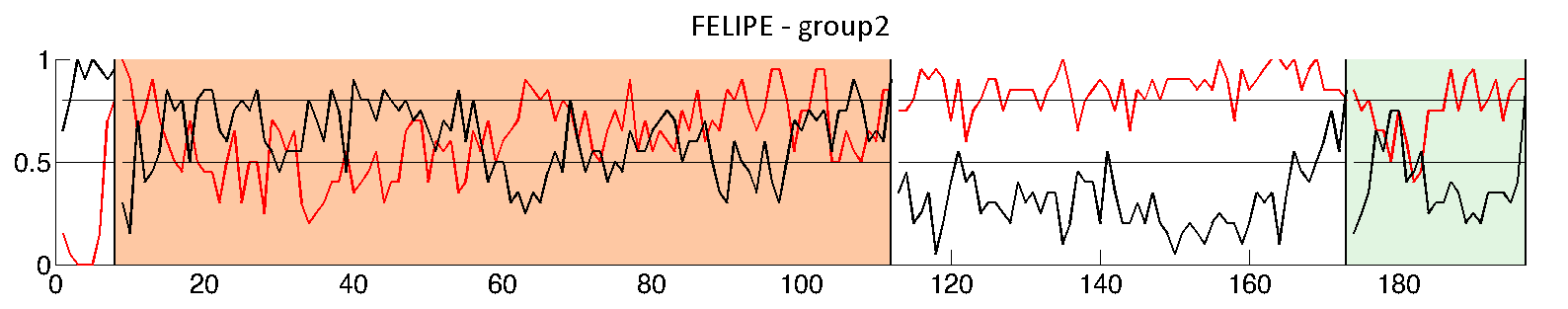


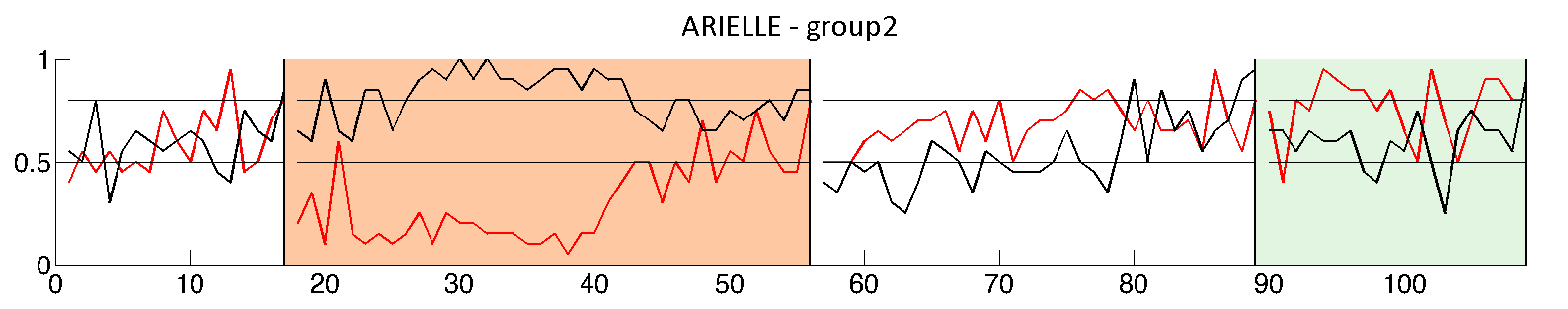

Supplement: Supplementary file 1 — Supplementary file1 (DOCX 346 kb) [file 13420_2022_522_MOESM1_ESM.docx]
